# Dental Infection Secondary to Acute Maxillary Sinusitis

**Published:** 1922-05

**Authors:** John A. Glassburg


### DENTAL INFECTION SECONDARY TO ACUTE MAXILLARY SINUSITIS

JOHN A. GLASSBURG, M.D.

### REPORT OF THREE CASES

The etiology of maxillary sinusitis includes the extension of infection from the teeth as one of the principal sources. The method of infection may be through direct continuity from a manifest or a hidden dental caries, a dead tooth, a periostitis, an osteitis of the alveolar process, a rupture of an infected dentigerous cyst, or through the circulation. Hajek, who is probably the most conservative in this particular, reports the dental origin at about eight per cent. Lermoyer and Luc place it at about fifty per cent. Tilley, going to the opposite extreme, estimates it at about 100 per cent. However, the general opinion is that the incidence of the dental etiology is from twenty to twenty-five per cent. Though all of these percentages are subject to much dispute, they are sufficiently strong to convince one that dental infection is an established and important cause of antrum disease. However guilty the teeth may be, there are occasions when they are not only innocent, but actually the victims of a primary infection of the maxillary sinus.

When dental caries or a periostitis is present, or an osteitis of the alveolar process, or a dentigerous cyst, the transference of the infection through direct continuity of the surface can be readily understood. However, in order fully to understand the conveyance of an infection through the circulation it is necessary to refer to the blood supply of the structures involved. Strubell found, on injecting the posterior alveolar arteries, three distinct groups of vessels; the first in the mucous membrane of the antrum (the periosteal layer), the second in the spongy bone of the maxilla, and the third in the covering of the alveolus and the roots of the teeth. These vascular groups, though showing individual characteristics, had so many connections and were so intimately anastomosed with one another that the three groups could be considered as really one system. It was thus shown that the blood supply of the antrum, the maxilla and the teeth was the same. Knowing the intimate communication of these three vascular groups, it is now easy to understand how the infection from the antrum can travel by way of the circulation to the teeth.

REPORT OF CASES

This condition, wherein the dental infection is secondary to a maxillary sinusitis, is illustrated by the cases here reported:

Case 1.—Agnes B., aged forty, complained of a severe neuralgic pain over the superior maxillary region and orbit on the left side. The pain was very acute, shooting in character and intermittent in type, and occurred every few minutes. The patient had not been able to sleep for three nights, and had been very restless during the daytime, continually applying hot poultices. This resulted in a marked swelling of the cheek and eyelids, but no relief of the pain.

Her dentist found nothing the matter with the teeth and referred her for a sinus examination. Rhinoscopy disclosed a hyperemia of the naris on the affected side, a hypertrophied mucous membrane, no septum deviation, no hypertrophied turbinates and no secretion. The patient was placed on conservative treatment consisting of hot nasal irrigations, hot tincture of benzoin compound inhalations, intestinal catharsis and an antipyretic potion, and referred for roentgenography. The roentgenograms revealed a definite shadow over the antrum, but no diseased condition of the teeth on the affected side.

The anterior end of the inferior turbinate and adjacent structures were anesthetized with ten per cent. cocain, and a needle puncture was made. Injection of physiologic sodium chlorid solution resulted in the evacuation of pus, which was mixed with the solution and emitted a fetid odor. About a quart of the solution was used until the return flow was clear. The patient felt much relieved and was told to return the following day, which, however, she did not do. She returned a month later complaining of similar symptoms, only milder in character. Rhinoscopy on this occasion revealed a condition similar to the one previously found. As a routine procedure the teeth were examined again, and this time tenderness was elicited over the second bicuspid. A roentgenogram revealed an abscess at the root, which had positively not been there on the previous examination. The tooth was extracted, and the patient made an uneventful recovery.

Case 2.—James H., aged twenty-eight, was referred by the family physician with a diagnosis of a probable pansinusitis with an acute maxillary antrum exacerbation. The patient complained of headache intensified by stooping, coughing or any jarring. The pain was general over the whole forehead, and was particularly severe over the left eye and left cheek bone. He gave a history of headache, nasal obstruction, and susceptibility to colds extending over a period of three years. This acute attack was of four days' duration.

Examination of the teeth detected no tenderness or manifest caries. Rhinoscopy revealed a septum moderately deviated to the left, a hypertrophied mucous membrane, and a polypoid middle turbinate on the left side. There was a thick secretion between the septum and the middle turbinate, and also between the turbinate and the lateral nasal wall. The diagnosis of pansinusitis of the left side was sustained, but because of the acuteness of the present condition, radical measures were postponed.

The maxillary sinus was punctured and irrigated, and pus was evacuated. Relief from the excruciating pain was almost instantaneous. The patient was then referred for roentgenography. The roentgen-ray findings were negative as far as the teeth were concerned, but showed shadows over the left frontal, the left ethmoid and the left maxillary sinus. The patient, who was a traveling salesman, had to leave and did not return for about three months, at which time he presented another exacerbation of the antrum infection. In addition, at this time the examination of his teeth disclosed an apical abscess of the first molar which had not been there previously. The antrum was punctured and irrigated, and at a later time the polypoid middle turbinate was removed, the ethmoid cells were exenterated, and the sphenoid sinus given a wide opening. The sinusitis was cleared up and the patient was referred to his dentist for the dental infection.

Case 3.—Henry G., aged thirty-two, came to the office complaining of a throbbing pain over the right side of the face. In addition he had a temperature of 101° F. The pain radiated over the cheek and the teeth to his ears and to his neck. He had first noticed this about three days before. Believing it to be a toothache, he went to his dentist, who removed a bridge from that side of the mouth, but found no focus of infection in the teeth and pronounced the teeth in good, healthy condition.

On further questioning it was ascertained that the patient was subject to frequent "colds in the head," and that at one time the trouble had been diagnosed as antral; but the symptoms had been so mild that he did not care to have any operation performed.

Rhinoscopy disclosed a hypertrophic rhinitis, but no secretion. The inferior turbinate and the adjacent structures were anesthetized. Pus was evacuated, and the washing with the physiologic sodium chlorid solution was continued until the return flow was clear. The patient was under treatment for two weeks. The acute symptoms subsided, but at each washing pus still showed. The secretion was thick, tenacious and foul and the needle opening was insufficient, it being blocked by the hypertrophied inferior turbinate. Removal of the anterior tip of the turbinate and enlargement of the opening were suggested, but as the patient was relieved of his subjective symptoms, he refused any further intervention.

He did not appear again until six months later, when he came, in great distress, the pain being even more severe than during the previous attack. The rhinoscopic picture was unchanged. As a routine procedure the teeth were examined, and this time tenderness was elicited over the second bicuspid and the first molar. Irrigation of the antrum revealed pus. As soon as the acute condition subsided, the anterior end of the enlarged turbinate was pushed away from the nasal wall and fractured. A better field being obtained, the antrum opening was well enlarged and free drainage was established. At the same time the patient was referred to his dentist, who this time reported abscesses of both the second bicuspid and the first molar teeth. The maxillary sinusitis was cleared up intranasally. At my final examination of him, he reported as still under the care of his dentist for the dental infection.

COMMENT

When these patients first appeared, all had sound teeth. This was satisfactorily demonstrated by the combined findings of three different men: the dentist, the roentgenologist and the rhinologist. Each of the patients had a definitely diagnosed acute maxillary sinusitis, which was conclusively proved by pus being obtained from every one of the antrums. In addition, one patient had a pansinusitis of the affected side. Each neglected the proper treatment of the antrum and developed another acute attack, at which time a recently developed dental infection was discovered in addition to the antrum infection already present. In view of the close anatomic relationship of the antrum of Highmore and the teeth involved in each case, it is reasonable and logical to conclude that the dental infection was due to the pus finding its way from the antrum to the teeth, and makes one believe that, if the proper attention had been given to the sinusitis at the right time, these teeth would in all probability have been saved.

